# Tuning the Intramolecular Chemiexcitation of Neutral Dioxetanones by Interaction with Ionic Species

**DOI:** 10.3390/molecules27123861

**Published:** 2022-06-16

**Authors:** Carla M. Magalhães, Joaquim C. G. Esteves da Silva, Luís Pinto da Silva

**Affiliations:** 1 Centro de Investigação em Química (CIQUP), Instituto de Ciências Moleculares (IMS), Faculdade de Ciências, Universidade do Porto, Rua do Campo Alegre s/n, 4169-007 Porto, Portugal; up201201533@edu.fc.up.pt (C.M.M.); jcsilva@fc.up.pt (J.C.G.E.d.S.); 2 LACOMEPHI, GreenUPorto, Departamento de Geociências, Ambiente e Ordenamento do Território, Faculdade de Ciências, Universidade do Porto, Rua do Campo Alegre s/n, 4169-007 Porto, Portugal

**Keywords:** bioluminescence, chemiluminescence, dioxetanone, electrostatic catalysis, thermolysis, chemiexcitation

## Abstract

The intramolecular chemiexcitation of high-energy peroxide intermediates, such as dioxetanones, is an essential step in different chemi- and bioluminescent reactions. Here, we employed the Time-Dependent Density Functional Theory (TD-DFT) methodology to evaluate if and how external stimuli tune the intramolecular chemiexcitation of model dioxetanones. More specifically, we evaluated whether the strategic placement of ionic species near a neutral dioxetanone model could tune its thermolysis and chemiexcitation profile. We found that these ionic species allow for the “dark” catalysis of the thermolysis reaction by reducing the activation barrier to values low enough to be compatible with efficient chemi- and bioluminescent reactions. Furthermore, while the inclusion of these species negatively affected the chemiexcitation profile compared with neutral dioxetanones, these profiles appear to be at least as efficient as anionic dioxetanones. Thus, our results demonstrated that the intramolecular chemiexcitation of neutral dioxetanones can be tuned by external stimuli in such a way that their activation barriers are decreased. Thus, these results could help to reconcile findings that neutral dioxetanones could be responsible for efficient chemi-/bioluminescence, while being typically associated with high activation parameters.

## 1. Introduction

Chemi- (CL) and bioluminescence (BL) occur in the conversion of thermal energy into excitation energy during a (bio)chemical reactions, with the emission of visible light [1,2,3]. As they do not require photo-excitation, CL and BL are associated with the diminished probability of autofluorescence arising from background signal [4,5]. So, these systems have been increasingly used in different areas, such as real-time imaging [6,7], sensing [8] and even in self-activating sensitizers in cancer therapy [9,10,11].

**Scheme 1:**
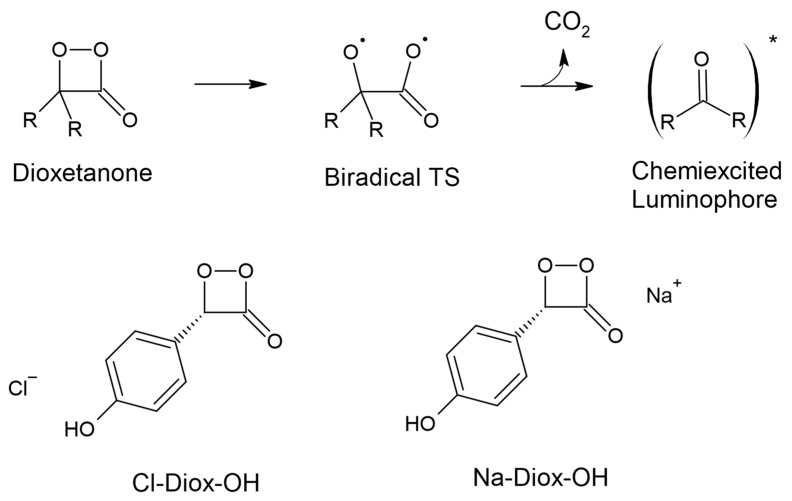
Schematic representation of the intramolecular chemiexcitation of dioxetanones (**top**). Schematic depiction of the systems here studied, which are composed by ionic species and a neutral dioxetanone species (**bottom**).

The light-emission from both CL and BL arises from a two-step process [12,13,14]. There is the initial oxygenation of the reactant, with subsequent generation and almost immediate thermolysis of an energy-rich peroxide intermediate (Scheme 1). It is this thermolysis reactions that allows for the thermally activated singlet ground state (*S*_0_) to be chemiexcited directly into the first singlet excited state (*S*_1_) of an oxidizable luminophore (Scheme 1). Dioxetanone is one of the most important types of these high-energy peroxide intermediates (Scheme 1), being found in several CL (as acridinium esters and 2-coumarones) and BL (as in fireflies, imidazopyrazinones and earthworms) systems [15,16,17,18,19,20].

Despite decades of research, the mechanism that controls the efficiency of singlet chemiexcitation is not yet understood, nor has it been agreed whether the ionization state of the dioxetanone is responsible for such efficient chemiexcitation. Given these factors, it is still difficult to develop CL/BL systems in a rational manner with enhanced and expected properties.

The Chemically Induced Electron-Exchange Luminescence (CIEEL) mechanism was the first to be employed in attempts to rationalize efficient singlet chemiexcitation [21]. It consists of electron transfer (ET) from an ionizable electron-rich group to the peroxide, followed by back ET (BET). The latter step is thought to be responsible for efficient singlet chemiexcitation due to charge annihilation. However, while initial reports indicated that model CIEEL systems were indeed efficient, subsequent re-evaluations revealed significantly lower quantum yields than expected [22,23].

Other authors tried to re-formulate the CIEEL theory in terms of more gradual charge transfer (CT) and back CT (BCT), between the ionizable electron-rich group and the peroxide moiety, typically involving anionic species. From this resulted the Charge Transfer-Initiated Luminescence (CTIL) mechanism [24,25]. However, theoretical and experimental studies indicated the possible involvement of neutral dioxetanones in efficient chemiexcitation, instead of anionic species [26,27,28,29,30]. More specifically, theoretical calculations made by our team revealed seemingly more beneficial chemiexcitation profiles [26,29,30]. In turn, Hirano et al. [27] performed an experimental and theoretical study of the chemiluminescence of *Cypridina* analogues and stated that the reaction mechanism of the chemiluminescence of neutral species should explain the highly efficient chemiexcitation of *Cypridina* bioluminescence. In another experimental/theoretical study of imidazopyrazinone molecules, Saito et al. [28] stated that their results could not validate expectations that electron-donating substituents on the phenyl group that are bound to the imidazopyrazinone core could enhance the singlet chemiexcitation yield. In fact, they stated that the CIEEL and CTIL mechanisms are not applicable for explaining the high singlet chemiexcitation yield in aequorin bioluminescence [28]. Furthermore, an analysis of different systems indicated that there was no clear relationship between CT/BCT and efficient singlet chemiexcitation, as some dioxetanones presented similar chemiexcitation profiles, despite their decomposition occurring with relevant differences in terms of CT/BCT [26,29,30,31,32].

Nevertheless, CIEEL and CTIL mechanisms are still being widely used to rationalize the chemiexcitation of different peroxide-based systems [3,12,18,24,25,33,34,35]. Indeed, CIEEL and/or CTIL could be operative in different CL/BL systems involving dioxetanones, because the *S*_0_ activation energy (Δ*E_act_*) for the thermolysis reaction of ET/CT-based dioxetanones tends to be significantly lower than for non-ET/CT-based ones (~10 vs. over ~20 kcal/mol) [18,24,25,26,27,28,29,30,31,32,33,34,35,36]. Furthermore, it should be noted that a lower Δ*E_act_* should be beneficial for CL/BL reactions, as it allows for higher light outputs [37]. Given this, if external effects could tune the neutral dioxetanones to undergo an ET/CT-based *S*_0_ thermolysis reaction, this could help to assess whether neutral dioxetanones could be responsible for efficient BL/CL, despite theoretical calculations describing them as having potentially too high Δ*E_act_* values [24,25,26,29,30,31,32].

Herein, we evaluated the hypothesis of if and how ionic species could catalyze the *S*_0_ thermolysis of a model neutral dioxetanone, by triggering ET and/or CT processes, while evaluating the possible changes to their singlet chemiexcitation profiles. To this end, we designed and modelled two different systems composed by an ion and the neutral dioxetanone species, in which ions of opposite charges were placed at different coordinates (Scheme 1). The neutral model dioxetanone was composed of the cyclic peroxide ring connected to an electron-rich moiety with an ionizable group (phenol group), due to the importance of ionizable electron-rich groups in both CIEEL and CTIL mechanisms. Furthermore, this model dioxetanone was recently studied by our team; therefore, we already know their thermolysis and chemiexcitation properties in the absence of the ions [36]. It should be noted that these models were not synthesized before (due to the difficulty of obtaining this kind of cyclic peroxides), which prevents the direct comparison between theoretical and experimental data. However, they can still be used as helpful models to understand the role of external stimuli on the chemiexcitation properties of dioxetanones.

The two model systems were as follows (Scheme 1): the first with the neutral dioxetanone and a chlorine anion (Cl^−^) placed near the hydroxyl group; the second with the neutral dioxetanone and a sodium cation (Na^+^) placed near the four-membered ring. The rationale for these systems is that the placement of ionic species could trigger ET/CT to better accommodate electrostatic interactions. More specifically, the addition of Cl^−^ near the phenol group was performed with the intention of triggering ET and/or CT from the phenol group to the peroxide, which should arise because of the necessity to reduce the negative charge in the phenol group due to the presence of a nearby anion. The addition of Na^+^ near the peroxide ring was performed with the aim of triggering ET and/or CT from the phenol to the peroxide, by increasing the negative charge in the four-membered ring for better accommodating electrostatic interactions with a nearby cation. In this study, we aimed to understand if neutral dioxetanones could be affected by external stimuli that can catalyze their S_0_ reaction via ET/CT, while maintaining similar singlet chemiexcitation profiles. This study was performing by employing the Time-Dependent Density Functional Theory (TD-DFT) approach. It should be noted that these complexes are theoretical models used to evaluate if and how external stimuli could affect the chemiexcitation properties of dioxetanones, and we do not claim that these complexes should be found in real CL/BL systems.

## 2. Results and Discussion

### 2.1. Study of the S_0_ Thermolysis Reaction

This work is divided into the following two sub-sections: a study of the *S*_0_ thermolysis reaction; and a study of the singlet chemiexcitation profile. As indicated before, we intended to evaluate if triggering ET and/or CT in neutral dioxetanones is possible, and what effects may occur as a result. To this end, we created two model systems including a phenol-bearing dioxetanone (Diox-OH), each with an ion with an opposite charge (Cl^−^ or Na^+^) placed at different vicinities of the dioxetanone (Scheme 1). These systems are termed Cl-Diox-OH and Na-Diox-OH (respectively, Scheme 1). It should be noted that the thermolysis and chemiexcitation of Diox-OH were already studied previously by our group [36].

**Figure 1:**
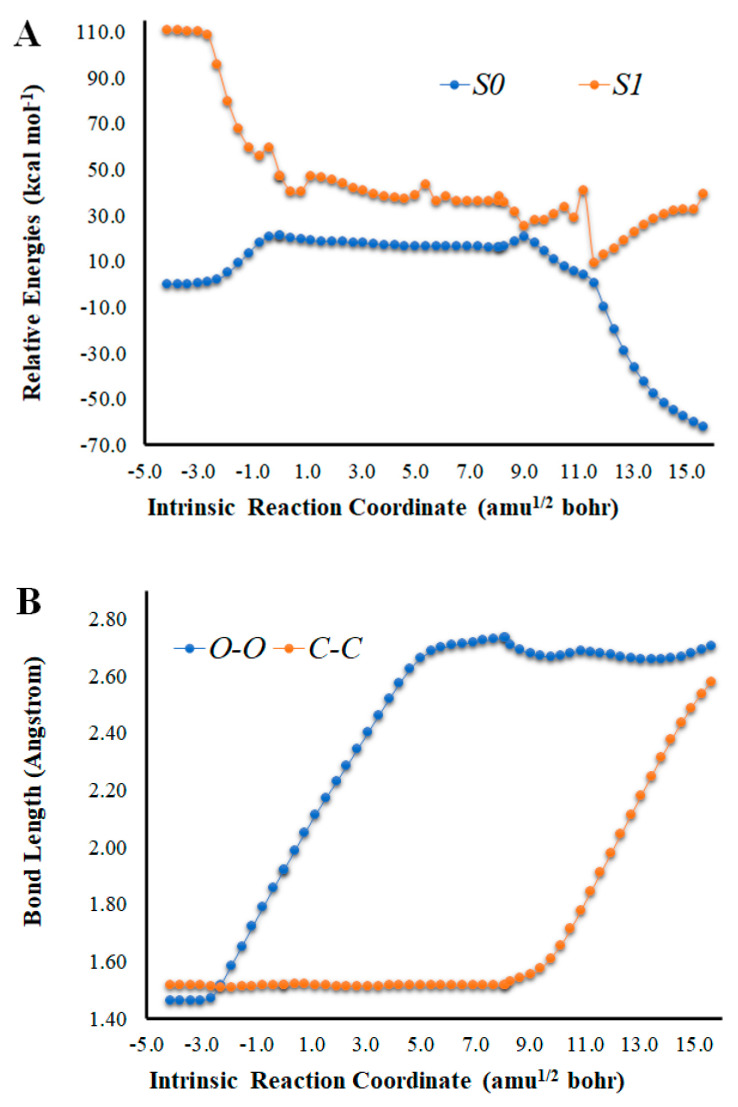

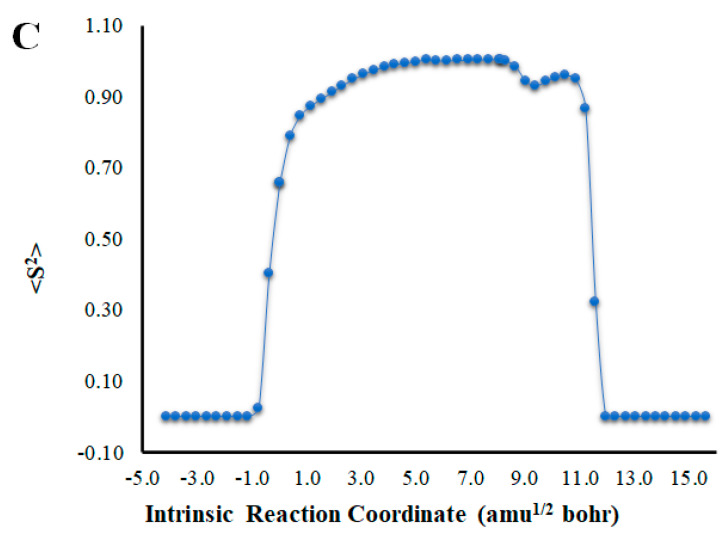
(**A**) Potential energy curves for the *S*_0_ and *S*_1_ states of Cl-Diox-OH as a function of the intrinsic reaction coordinates. (**B**) Bond length variation of O-O and C-C bonds of the four-membered cyclic ring of Cl-Diox-OH. (**C**) Determination of <*S*^2^> for Cl-Diox-OH, as a function of intrinsic reaction coordinates.

**Figure 2:**
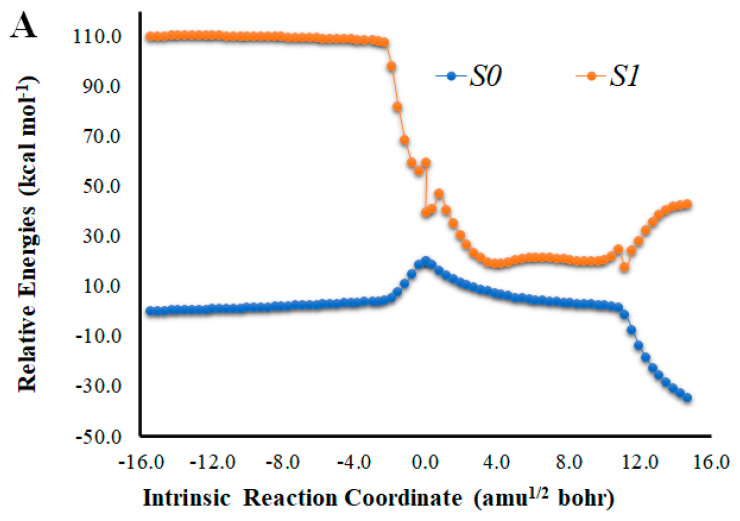

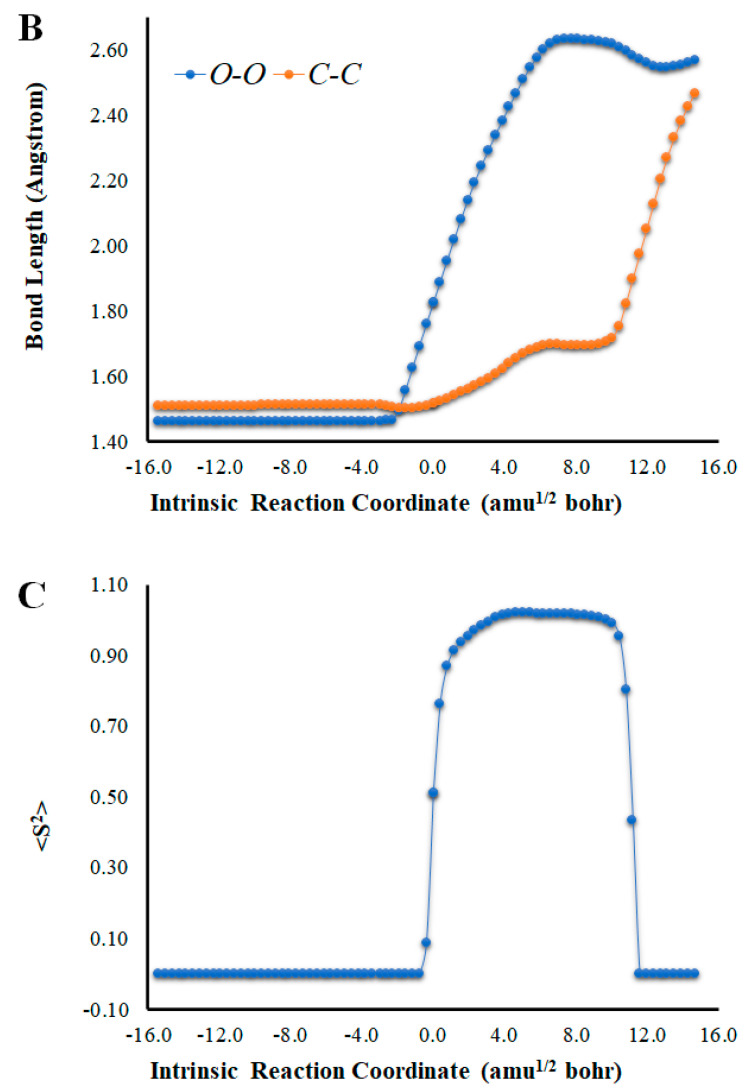
(**A**) Potential energy curves for the *S*_0_ and *S*_1_ states of Na-Diox-OH as a function of the intrinsic reaction coordinates. (**B**) Bond length variation of O-O and C-C bonds of the four-membered cyclic ring of Na-Diox-OH. (**C**) Determination of <*S*^2^> for Na-Diox-OH, as a function of intrinsic reaction coordinates.

The potential energy curves for the *S*_0_ thermolysis of Cl-Diox-OH and Na-Diox-OH are shown in Figure 1 and Figure 2 (respectively), as a function of intrinsic reaction coordinates. The calculated Δ*E_act_* for the *S*_0_ thermolysis reactions are of 20.9 kcal mol^−1^ for Cl-Diox-OH, and of 19.9 kcal mol^−1^ for Na-Diox-OH. Quite interestingly, the Δ*E_act_* for Diox-OH was of 24.2 kcal mol^−1^ [36], meaning that the introduction of the ions did lead to a decrease of 3.3 and 4.3 kcal mol^−1^ for the Δ*E_act_* of Cl-Diox-OH and Na-Diox-OH, respectively. More importantly, while the Δ*E_act_* for Diox-OH might be associated with a very slow CL/BL emission [38,39], not in line with experimental data for efficient CL/BL systems, the Δ*E_act_* for the ionic systems were in line with a flash-type emission profile typically observed for different CL/BL systems [38,39]. Thus, the inclusion of the ions was expected to lead to relevant changes in the thermodynamic properties of these systems. Given this, our next step was to evaluate if this decrease in Δ*E_act_* was due to the triggering of ET and/or CT steps.

An analysis of the key geometrical parameters for the thermolysis of dioxetanones, the bond lengths of the O-O and C-C bonds of the peroxide ring (Scheme 1) [18,24,25,26,27,28,29,30,31,32,33,34,35,36], revealed that Cl-Diox-OH (Figure 1) follows the typical stepwise pathway that consists of initial O-O bond breaking, followed by C-C bond stretching [18,24,25,26,27,28,29,30,31,32,33,34,35,36]. As for Na-Diox-OH (Figure 2), while O-O and C-C bond breaking is not simultaneous, these processes do appear to be concerted. The determination of <*S*^2^> values of ~1.0 for both Cl-Diox-OH (Figure 1) and Na-Diox-OH (Figure 2) reveals that these thermolysis reactions proceed via the formation of a biradial species. These findings are in line with data found for dioxetanones in general [18,24,25,26,27,28,29,30,31,32,33,34,35], and for Diox-OH specifically [36]. Thus, the inclusion of the two ions did not affect the thermolysis reaction mechanism.

**Figure 3:**
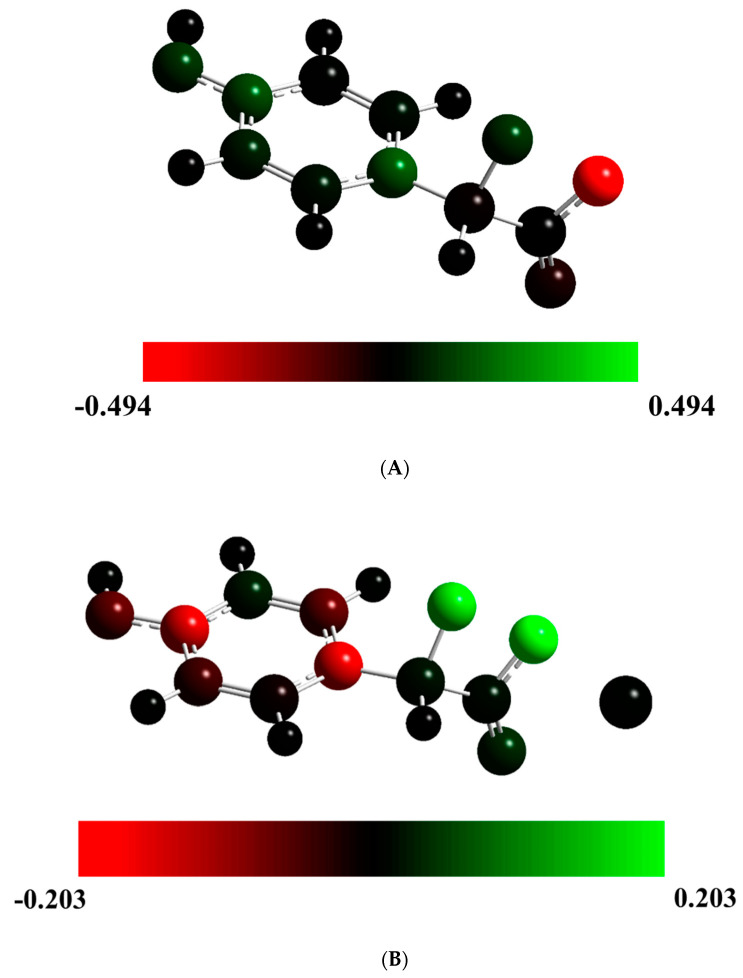
Mulliken spin density for Cl-Diox-OH (**A**) and Na-Diox-OH (**B**) at their TS structures, represented with color atoms by density.

To evaluate the potential trigger of ET by the ionic species, we present, in Figure 3, the Mulliken spin density for the TS structures of Cl-Diox-OH and Na-Diox-OH. It should be noted that for Diox-OH [36], the spin density was located solely on the two oxygen heteroatoms that composed the peroxide bond, indicative of the biradical being formed due to homolytic O-O bond breaking (typical of neutral dioxetanones). Interestingly, the Mulliken spin density of Cl-Diox-OH was delocalized between one of the two oxygen heteroatoms of the peroxide and the phenol moiety, indicating that placing a Cl^−^ species does trigger ET from the electron-rich moiety to the peroxide (thereby introducing CIEEL character to the *S*_0_ thermolysis). The Mulliken spin density is somewhat different for Na-Diox-OH, as both oxygen heteroatoms that constituted the peroxide bond possess spin density. Nevertheless, they are of the same spin, with the spin density of the opposite sign being placed in the phenol moiety. Therefore, the placement of the Na^+^ species also induces CIEEL characteristics in the *S*_0_ thermolysis of Na-Diox-OH.

**Figure 4:**
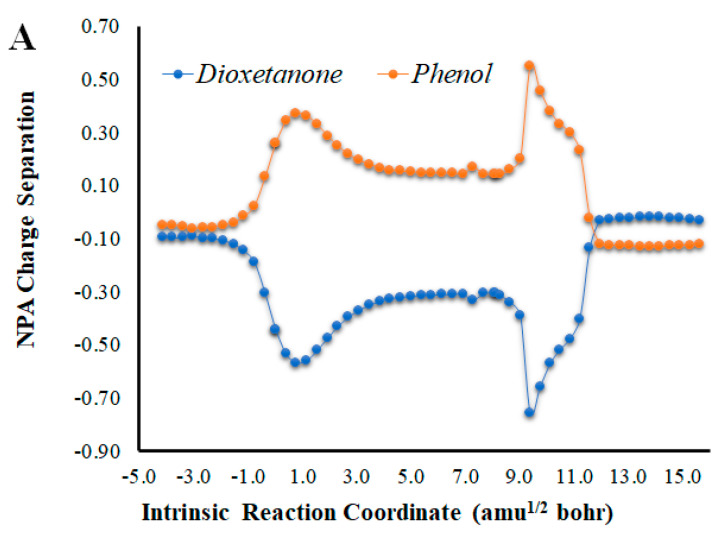

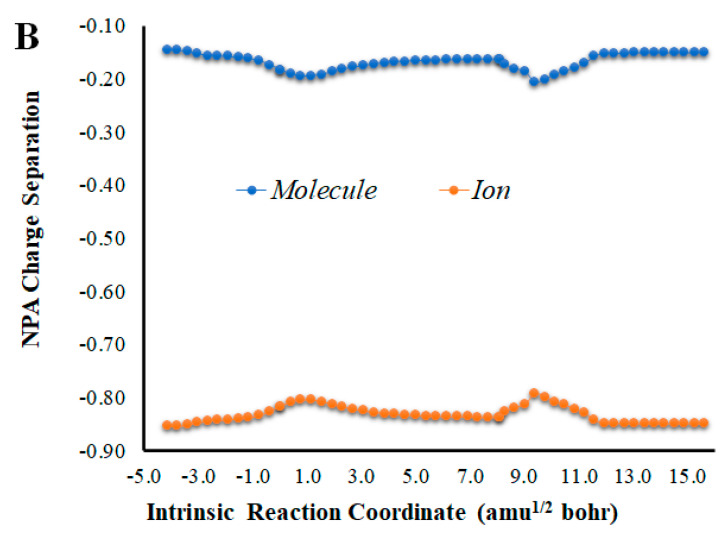
(**A**) NPA charge separation between the dioxetanone and phenol moieties of *S*_0_ Cl-Diox-OH, as a function of intrinsic reaction coordinates. (**B**) NPA charge separation between the dioxetanone molecule and Cl^−^ ion of *S*_0_ Cl-Diox-OH, as a function of intrinsic reaction coordinates.

**Figure 5:**
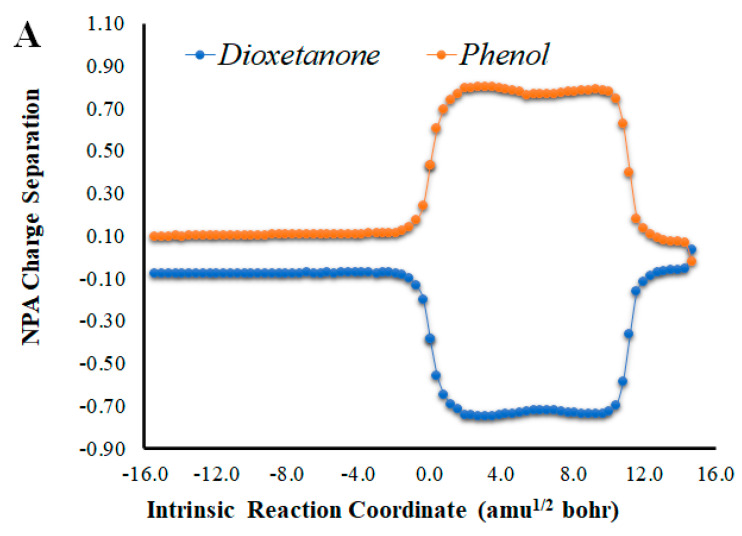

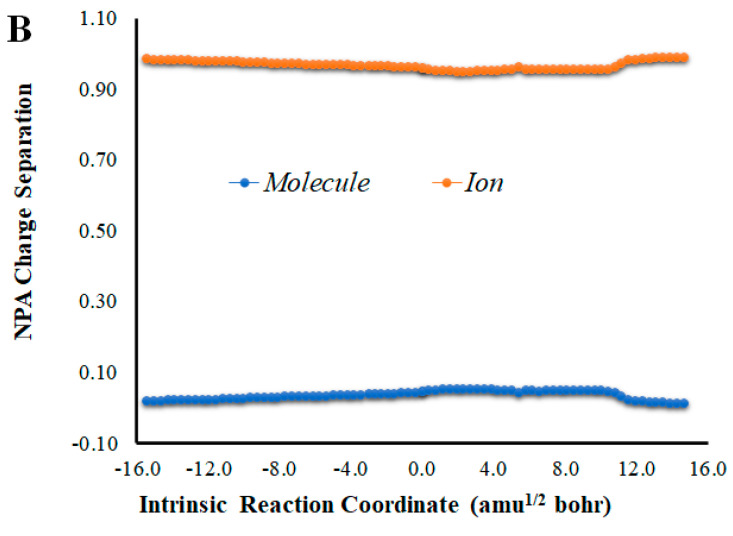
(**A**) NPA charge separation between the dioxetanone and phenol moieties of *S*_0_ Na-Diox-OH, as a function of intrinsic reaction coordinates. (**B**) NPA charge separation between the dioxetanone molecule and Na^+^ ion of *S*_0_ Na-Diox-OH, as a function of intrinsic reaction coordinates.

Given that placing the ionic species in the vicinities of Diox-OH triggered ET from the phenol to the peroxide, we also evaluated if those species could induce CT. To that end, we calculated the NPA charge separation between the phenol and cyclic peroxide moieties for the two systems (Figure 4 and Figure 5) as a function of intrinsic reaction coordinates. It should be noted that the thermolysis of Diox-OH proceeded without relevant CT [36]. In contrast to Diox-OH, the thermolysis of Cl-Diox-OH proceeds with relevant CT in the vicinity of the biradical TS, associated with peroxide bond breaking, with a charge separation between the dioxetanone and phenol moieties of 0.94*e* (Figure 4). Thus, placing a Cl^−^ species near the phenol also triggers relevant CT during peroxide bond breaking, providing CTIL characteristics to this reaction. The same can be said for Na-Diox-OH (Figure 5), as an even higher charge separation (of ~1.5*e*) was found. Interestingly, the charge separation profiles present relevant differences between systems. For Cl-Diox-OH (Figure 4), we observed an initial CT in the vicinity of the TS, with a return to somewhat basal charge separation values until the rupture of the peroxide was reached, in which further CT/BCT was achieved. This latter CT/BCT, associated with final peroxide rupture, is not uncommon for neutral dioxetanones [36]. However, in the case of Na-Diox-OH, there is an initial CT with peroxide bond breaking at the TS, but subsequent BCT only occurs with peroxide ring rupture (Figure 5). This profile is more typical of CTIL-based anionic dioxetanones [36]. We also evaluated the charge separation between the dioxetanone molecule and the ion for both Cl-Diox-OH (Figure 4) and Na-Diox-OH (Figure 5). While the presence of the ion does trigger CT within the dioxetanone molecule, relevant CT was not observed between the ion and the molecule for either case.

In summary, our results showed that there is indeed the possibility that external stimuli can catalyze the *S*_0_ thermolysis of neutral dioxetanones. More specifically, the strategic placement of ionic species triggered ET and CT between the electron-rich moiety and the peroxide ring of a neutral dioxetanone. By providing CIEEL and CTIL characteristics to these reactions, the *S*_0_ Δ*E_act_* of these species were in line with an emissive flash profile, which is typically associated with different CL/BL systems.

### 2.2. Study of Singlet Chemiexcitation Profiles

After obtaining evidence that nearby ionic species can trigger ET and CT processes that reduce the Δ*E_act_* for the *S*_0_ thermolysis of neutral dioxetanones, we proceeded to evaluate the singlet chemiexcitation profile of both Cl-Diox-OH and Na-Diox-OH. The potential energy curves for both *S*_0_ and *S*_1_ regarding Cl-Diox-OH and Na-Diox-OH are presented in Figure 1 and Figure 2, respectively.

First, it should be noted that for Diox-OH, singlet chemiexcitation occurs in a large and flat biradical region with a length of 10.7 am^1/2^ bohr, in which *S*_0_ and *S*_1_ are nearly degenerated/degenerated with energy gaps of 6.0–11.5 kcal mol^−1^ [36]. This type of profile can be associated with near-infinite possibilities for non-adiabatic transitions [20,24,26,29,30,31,32,33]. Furthermore, multireference calculations should predict smaller energy gaps, due to an energy error in this region by the TD-DFT approach resulting from spin contamination in the reference state using BS technology [20,24,26,29,30,31,32,33]. Nonetheless, TD-DFT calculations have been extensively shown to provide accurate qualitative results for dioxetanones and other peroxides [20,24,26,29,30,31,32,33].

The analysis of the *S*_0_ and *S*_1_ potential energy curves for Cl-Diox-OH (Figure 1) shows that the introduction of a Cl^−^ species into the system does affect its singlet chemiexcitation profile. While the *S*_0_ biradical PEC region is indeed flat to a significant extent (length of ~10 amu^1/2^ bohr), the *S*_0_ and *S*_1_ are not as degenerated in that region as in the case of Diox-OH [36]. That is, except for three values of coordinates, the *S*_0_–*S*_1_ energy gap is always higher than 13.0 kcal mol^−1^. Nevertheless, Cl-Diox-OH still presents three values of coordinates in which the *S*_0_–*S*_1_ energy gap is relevantly low, which are as follows: 4.9 kcal mol^−1^ at 9.0 amu^1/2^, 10.3 kcal mol^−1^ at 9.4 amu^1/2^ bohr, and 8.7 kcal mol^−1^ at 11.6 amu^1/2^ bohr.

The introduction of a Na^+^ species nearby the peroxide ring also affects the singlet chemiexcitation profile of Na-Diox-OH (Figure 2). Interestingly, while the *S*_0_ biradical region also extends for a significant length (~11 amu^1/2^ bohr), it is no longer flat. This lack of “flatness” could be attributed to the full CTIL character presented by Na-Diox-OH (Figure 5), as previous studies found that access to the large and flat region of the PEC where *S*_0_ and *S*_1_ significantly degenerated could be prevented by CTIL-based decomposition [40]. In fact, the *S*_0_ thermolysis of Cl-Diox-OH proceeds via an initial CTIL-like process in the vicinity of the TS (Figure 4) but stops during the remaining part of the biradical region, thereby not being a full CTIL-based decomposition. Regarding the singlet chemiexcitation profile, there is not as large a region of degeneracy between states as found for Diox-OH [36]. There is only a smaller region of the PEC (between values of coordinates of 2.7 and 5.0 amu^1/2^ bohr) in which the *S*_0_–*S*_1_ energy gaps are of 11.8–14.8 kcal mol^−1^ (Figure 2).

Given these results, the introduction of ionic species near the neutral dioxetanone does affect the singlet chemiexcitation profile of Diox-OH, probably due to triggering ET and CT processes during the thermolysis reaction. However, it should be noted that we also previously studied the singlet chemiexcitation profile of Diox-OH with a deprotonated phenol moiety (Diox-O^−^) [36]. This dioxetanone decomposes with clear and full CIEEL and CTIL characteristics [36]. Interestingly, the *S*_0_–*S*_1_ energy gaps were always higher than 17.1 kcal mol^−1^, except for three values of coordinates [36]. However, even at those coordinate values, the energy gaps were still high, in the range of 15.5–16.4 kcal mol^−1^. More importantly, these energy gaps were higher than the ones found for both Cl-Diox-OH (lower ones of 4.9–10.3 kcal mol^−1^) and Na-Diox-OH (lower ones of 11.8–14.8 kcal mol^−1^). Given this, the singlet chemiexcitation profile of Cl-Diox-OH and Na-Diox-OH could be at least comparable with the one presented by the full CIEEL/CTIL-based Diox-O^−^.

In summary, we determined that the introduction of nearby ionic species does decrease the efficiency of the singlet chemiexcitation of these systems, when comparing with the neutral model dioxetanone. However, their chemiexcitation profiles do not appear to be less beneficial than that of anionic dioxetanones. Thus, it can be stated that while ionic species impact singlet chemiexcitation, probably by triggering ET/CT, their effects should not prevent the singlet chemiexcitation of neutral dioxetanones.

## 3. Theoretical Methods

The TD-DFT methodologies employed here are based on a previously used approach in studies performed by our group [9,11,20,26,29,30,31,32,36]. *S*_0_ geometry optimizations and frequency calculations of the two studied systems (Scheme 1) were made with the ωB97XD density function [41]. We employed a closed-shell approach (R) for reactants and products. Meanwhile, an open-shell (U) was used for transition states (TSs) along with broken-symmetry technology to make an initial guess for a biradical. IRC calculations were performed to ensure that the TS connected with the expected reactants and products. The cartesian coordinates for the TSs can be found in Tables S1 and S2. The geometry optimization, frequency and IRC calculations were performed with the 6-31G(d,p) basis set for H, C, O and Na atoms, while the LANL2DZ basis set was used for the Cl atom.

Subsequently, the *S*_0_ energies for the structures obtained with IRC calculations were re-evaluated using single-point calculations with the same functional as before (ωB97XD), while increasing the basis sets. Specifically, we used the 6-31+G(d,p) basis set for H, C, O and Na atoms, and the basis set LANL2DZ with polarization and diffuse functions for Cl. The *S*_1_ energies were obtained for each IRC-obtained structure with a TD-DFT approach, by single-point calculations on top of *S*_0_ structures with the same level of theory used for the re-evaluation of *S*_0_ energies.

ωB97XD was the chosen density functional as it provides accurate estimates for π → π* and n → π* local excitations, and CT and Rydberg states [42]. Furthermore, long-range-corrected hybrid exchange-correlation functionals (including ωB97XD) are known to provide accurate results for the chemiexcitation of CL/BL-capable peroxides [9,11,20,24,25,26,29,30,31,32,36].

All calculations were performed in vacuo to better compare the results obtained previously for the neutral model dioxetanone in the absence of nearby ions [36]. Furthermore, we focused on the intrinsic effects of the ions on the chemiexcitation of this dioxetanone, without being affected by solvent effects. Finally, all TD-DFT/DFT calculations were made with the Gaussian 09 program package [43].

## 4. Conclusions

Here, we used a TD-DFT methodology to assess if external stimuli could tune the singlet chemiexcitation step of neutral model dioxetanones in a way that decreased the activation barrier of the *S*_0_ thermolysis reaction, while retaining similar singlet chemiexcitation profiles. More specifically, we investigated if the strategic placement of nearby ionic species could trigger ET and/or CT processes during the *S*_0_ thermolysis, without affecting the typical *S*_0_ → *S*_1_ chemiexcitation transitions of neutral dioxetanones.

Our data revealed that the nearby ionic species were indeed able to trigger both ET and CT processes during peroxide bond breaking, which decreased the measured Δ*E_act_* to values low enough to be compatible with the emissive flash-profile associated with different CL/BL systems. Nevertheless, the inclusion of these species affected the singlet chemiexcitation profiles of these systems, as compared to the neutral dioxetanone model. However, these profiles do not appear to be less efficient than that of anionic model dioxetanone.

In summary, our results showed that the intramolecular chemiexcitation of neutral dioxetanones can be tuned by external stimuli in such a way that their Δ*E_act_* can be reduced while retaining similar chemiexcitation profiles. These results can help to reconcile findings that neutral dioxetanones could be responsible for efficient CL/BL, while being typically associated with a very high Δ*E_act_*.

## Data Availability

Not applicable.

## Supplementary Materials

The following supporting information can be downloaded at: https://www.mdpi.com/article/10.3390/molecules27123861/s1, Table S1: Cartesian coordinates for the TS structure of Cl-Diox-OH, Table S2: Cartesian coordinates for the TS structure of Na-Diox-OH.

## Sample Availability

Not available.
