# Supplementary material for: Tuning the Intramolecular Chemiexcitation of Neutral Dioxetanones by Interaction with Ionic Species
Source: Molecules. 2022 Jun 16;27(12):3861. doi: 10.3390/molecules27123861 (PMC9228516; doi:10.3390/molecules27123861)
Supplement: Supplementary file 1 [file molecules-27-03861-s001.zip › molecules-1774112-supplementary.pdf]

Tuning the Intramolecular Chemiexcitation of Neutral Dioxetanones by Interaction with Ionic Species

Carla M. Magalhães [1], Joaquim C. G. Esteves da Silva [1,2] and Luís Pinto da Silva [1,2,*]

[1] Centro de Investigação em Química (CIQUP), Instituto de Ciências Moleculares (IMS), Faculdade de Ciências, Universidade do Porto, Rua do Campo Alegre s/n, 4169-007 Porto, Portugal; up201201533@edu.fc.up.pt (C.M.M.); jcsilva@fc.up.pt (J.C.G.E.d.S.)

[2] LACOMEPHI, GreenUPorto, Departamento de Geociências, Ambiente e Ordenamento do Território, Faculdade de Ciências, Universidade do Porto, Rua do Campo Alegre s/n, 4169-007 Porto, Portugal

* Correspondence: luis.silva@fc.up.pt

Table S1. Cartesian coordinates for the TS structure of Cl-Diox-OH.

O	-0.03427	2.61160	-1.60740
C	0.70045	2.46658	-0.43118
C	2.06165	2.44786	-1.10196
O	1.73405	2.76678	-2.34094
O	3.18662	2.26352	-0.70627
C	0.10029	1.25060	0.17746
C	-0.72344	1.34611	1.31727
C	-1.32620	0.23064	1.84590
C	-1.15197	-1.03810	1.23281
C	-0.34967	-1.13742	0.06426
C	0.24183	-0.00991	-0.45017
H	0.62282	3.33026	0.24832
H	-0.86418	2.31550	1.78871
H	-1.94378	0.28385	2.73595
H	-0.23547	-2.11864	-0.38928
H	0.84123	-0.07970	-1.35311
O	-1.74930	-2.06335	1.78003
H	-1.56858	-2.94227	1.26608
Cl	-1.19079	-4.48113	0.28012

Table S2. Cartesian coordinates for the TS structure of Na-Diox-OH.

O	-0.34452	1.37506	-1.46125
C	0.02797	1.27257	-0.10320
C	1.50054	1.19611	-0.44971
O	1.45658	1.47854	-1.72723
O	2.53529	0.99428	0.16016
C	-0.74593	0.11777	0.41001
C	-1.67556	0.27533	1.45970
C	-2.41753	-0.79318	1.89939
C	-2.26891	-2.05012	1.27290
C	-1.36775	-2.21533	0.20332
C	-0.62799	-1.13872	-0.22742
H	-0.17266	2.19089	0.46377
H	-1.79409	1.24510	1.93242
H	-3.12348	-0.70190	2.71640
H	-1.26778	-3.18194	-0.28080
H	0.04849	-1.25082	-1.06832
O	-3.02096	-3.03808	1.74459
H	-2.87522	-3.86030	1.26305
Na	3.92085	1.42137	-1.65781
